# Improvement of the fuel properties of dairy manure by increasing the biomass-to-water ratio in hydrothermal carbonization

**DOI:** 10.1371/journal.pone.0269935

**Published:** 2022-07-18

**Authors:** Mohammed Aliyu, Kazunori Iwabuchi, Takanori Itoh

**Affiliations:** 1 Graduate School of Agriculture, Hokkaido University, Kita-ku, Sapporo, Hokkaido, Japan; 2 Agricultural and Bioresources Engineering, Federal University of Technology, Minna, Niger State, Nigeria; 3 Research Faculty of Agriculture, Hokkaido University, Kita-ku, Sapporo, Hokkaido, Japan; 4 Tanigurogumi Corporation, Nasushiobara, Tochigi, Japan; Universiti Malaysia Pahang, VIET NAM

## Abstract

There are many advantages to liquid-based hydrothermal carbonization (L-HTC) but the need to immerse the biomass in water generates more post-process water, hindering the commercialisation of HTC. To address this issue, this study investigated the feasibility of vapour-based HTC (V-HTC), which minimizes the water required. Dairy manure was hydrothermally treated at temperatures of 200, 230, 255 and 270°C and biomass-to-water ratios (B/W) of 0.1, 0.18, 0.25, 0.43, 0.67 and 1.0 for 20 minutes, then the produced hydrochars were characterized by calorific, proximate, ultimate and thermogravimetric analyses. The results showed that the mass yields of hydrochar decreased with increasing temperature but were essentially stable at high B/W ratios. Notably, the calorific values of the hydrochars increased with increasing temperature and B/W ratio, and the energy density increased by 46%. Due to the higher mass yield and increased energy density, maximum energy yields at each temperature (86.0–97.4%) were observed at a B/W ratio of 1.0. The proximate and ultimate analyses revealed that the degree of coalification, such as the increase in carbon content and decrease in oxygen and volatile matter, progressed more under V-HTC than L-HTC conditions, likely because the lower liquid content in V-HTC facilitates the formation of secondary char and increases the reaction severity due to higher acidity. This study showed a potential approach for upgrading a semi-solid-state biomass by V-HTC.

## Introduction

The consumption of fossil fuels as energy sources has raised many global concerns because of their negative impact on the environment, such as greenhouse gas emissions [1, 2]. The utilization of biomass as an alternative energy source is thus imperative due to its carbon-neutral nature and sustainability. Billions of tons of biomass such as animal manure are generated annually, and animal husbandry is increasing rapidly globally. Hence, animal manure provides a sustainable raw material for biofuel production, but biomass typically must be pre-treated for efficient use as a solid biofuel [3]. Hydrothermal carbonization (HTC) is a biomass low energy consumption pre-treatment technique. This effective thermochemical approach converts biomass into value-added carbon-rich products [3, 4]. The solid product generated from HTC is usually referred to as hydrochar. Hydrochar has enormous potential application in energy generation, and thus much research has focused on the production of high-quality hydrochar using HTC [3–8].

Compared to other available thermal conversion technologies for biomass, HTC is better suited for converting biomass with high moisture content, such as livestock manure, because it uses moderately hot compressed water between 180 to 280°C in an autoclave reactor [4, 9–12]. However, the need to use water, which is a unique feature of HTC, requires proper wastewater treatment after the HTC process to remove dissolved organic acids and other compounds prior to discharge into the environment [3, 4]. In the most commonly used HTC process, called liquid-based HTC (L-HTC), the feedstock is immersed in sufficient water to facilitate higher heat exchange between the feedstock and the liquid medium, [13, 14] but this tends to generate more post-process wastewater [5]. Attempts to address this include water recycling, recovery of dissolved organic chemicals, usage in anaerobic digestion processes, and wastewater treatment, [3, 15–19] but all add significant additional construction and operating costs to commercial scale operations.

To address these issues, the present study considers the feasibility of vapour-based HTC (V-HTC), which is easily achieved by increasing the biomass-to-water (B/W) ratio. The feedstock is moist or has no contact with bulk liquid water [13, 14]. Instead, biomass decomposition is mainly governed by water vapour, which can penetrate the biomass matrix faster due to its lower density compared to liquid [5]. V-HTC thus holds promise for simplify the handling of post-process water by avoiding excessive water usage prior to HTC treatment and/or requiring little mechanical dewatering after HTC treatment while improving fuel properties similar to that achieved by L-HTC.

The transition from L-HTC to V-HTC by varying the B/W ratio implies that the heat transfer medium leading to biomass decomposition is either liquid, vapour, or a mixture of the two [20]. Thus, the fuel properties of hydrochar are likely influenced by the B/W ratio but there is little direct evidence to support this. Several studies have shown that the B/W ratio has little effect on mass and energy yields [21, 22], whereas other studies have found that increasing the B/W ratio in HTC increases the mass yield and calorific value of hydrochar [23–25]. For instance, Volpe and Fiori [23] found that the higher heating value (HHV) of hydrochar from olive pulp increased from 26.6 to 28.4 MJ/kg, achieved by increasing the B/W ratio from 0.08 to 0.25. Also, Aragón-Briceño et al. [26] investigated the effect of increasing the solids loading from 2.5% to 30% (equivalent to a B/W of 0.03 to 0.43) on the characteristics of hydrochar. The study found that the HHV improved from 14.4 to 16.5 MJ/kg. Importantly, to our knowledge, there are no published studies on HTC for B/W ratios above 0.67. Thus, HTC using feedstock with a moisture content below 60 wt.% (corresponding to a B/W ratio above 0.67) may be of limited utility, or perhaps water must be added to perform HTC. However, the moisture content of feedstock varies greatly depending on the situation and can be below 60 wt.% in some cases [12], and thus HTC using a B/W ratio above 0.67 deserves investigation. HTC is conducted in a tightly closed system, ensuring that the generated vapour is in continuous contact with the feedstock, allowing hydrochar production at higher B/W ratios. The authors therefore proposed the following hypothesis: hydrochar with improved fuel properties can be produced from low moisture content biomass without requiring the addition of more water. To verify this hypothesis, dairy manure (DM) as a test feedstock was hydrothermally treated at B/W ratios of 0.1, 0.18, 0.25, 0.43, 0.67 and 1.0, the calorific values were measured, and proximate, ultimate and thermogravimetric analyses were conducted to evaluate the fuel properties of the resulting hydrochars.

## Materials and methods

### Materials

The DM used as a test material was obtained from an experimental farm at the Field Science Centre for Northern Biosphere, Hokkaido University, Japan. The sample was dried in an electric oven for 24 hours at 105°C to a constant mass, then crushed using a mortar and pestle to reduce the particle size for ease of storage in a sealed Ziploc plastic bag [10, 27]. The sample was stored prior to the experiment.

### Classification of the HTC process by the degree of saturation

There is no clear definition of the classification state of HTC and thus the present study used the degree of saturation (*S*_*r*_ [%]), which is the ratio of water to the void fraction on a volumetric base, to classify four HTC states: complete L-HTC (*S*_*r*_ = 100%), predominance of L-HTC (67%≤*S*_*r*_<100%), mixture of liquid and vapour (33%≤*S*_*r*_<67%), and predominance of V-HTC (0%≤*S*_*r*_<33%). Even samples classified as predominance of L-HTC or V-HTC likely involve the action of vapour or liquid to some extent, respectively. *S*_*r*_ was calculated using Eq (1):

$$S_{r}=\frac{V_{w}}{V_{a}+V_{w}}\times 100$$ (1)

where *V*_w_ (m^3^) and *V*_a_ (m^3^) are the volumes of water and air, respectively, and were determined using Eqs (2) and (3):

$$V_{w}=\frac{m_{w}}{\rho_{w}}$$ (2)

$$V_{a}=V-V_{w}-\frac{m_{b}}{\rho_{b}}$$ (3)

Here, *m*_*w*_ (kg) and *ρ*_w_ (kg/m^3^) are the mass and specific density of water; *V* (m^3^) is the apparent volume of the prepared sample in the reactor; and *m*_b_ (kg) and *ρ*_b_ (kg/m^3^) are the dry mass and specific density of DM, respectively. Note that the specific density of DM (*ρ*_b_) was determined to be 1.9 using a pycnometer at room temperature.

### HTC reactor set-up and procedure

**Fig 1:**
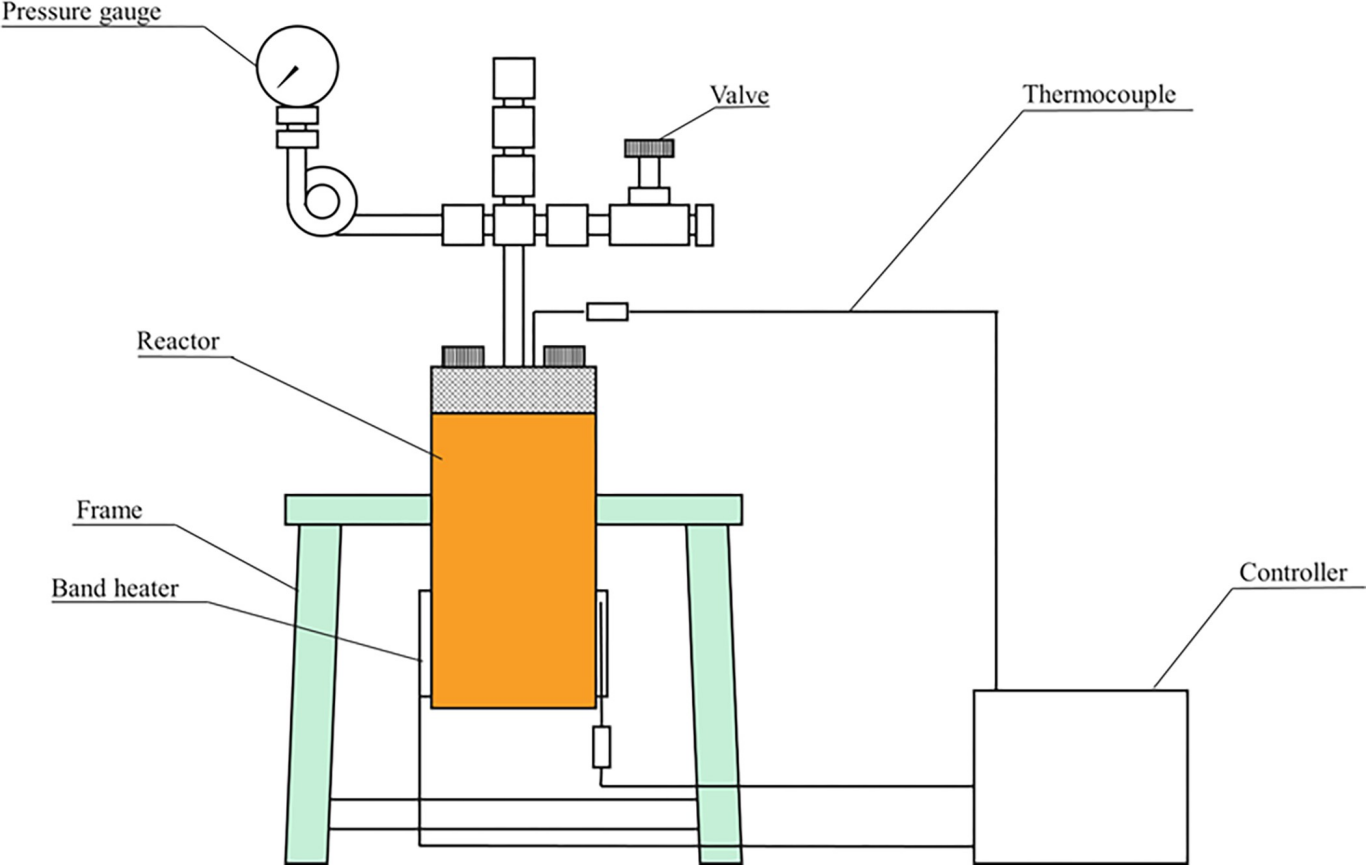
Schematic of the carbonization reactor used in this study.

Experiments were performed in a 70 mL stainless steel TVS-N2 (Taiatsu Techno, Tokyo, Japan) batch reactor (Fig 1) with a temperature and pressure limit of 300°C and 8 MPa.

Raw feedstocks were prepared by mixing about 5 to 20 ± 0.01 g of oven-dried sample and 20 to 45 ± 0.01 g of deionized water to obtain the desired B/W ratio with high accuracy (Eq [4]).


$$B/W\;ratio=\frac{m_{b}}{m_{w}}$$ (4)

For each batch experiment, prepared raw feedstock was placed in the reactor and sealed, then flushed three times with pure nitrogen to remove oxygen. The process temperature range was 200 to 270°C and the holding time was 20 minutes. A PID controller was used to achieve the desired temperature. The process was initiated at atmospheric pressure, and the average pressure (autogenic) rise in the reactor ranged from 1.5 to 6.2 MPa. After terminating the reaction, the reactor was placed in cold water for rapid cooling. For the L-HTC process, the liquid and hydrochar were separated by filtration and the liquid was discarded. For the V-HTC process, no aqueous phase was visible, and thus the sample was not filtered. The recovered hydrochar was dried to a constant mass in an electric oven at 105°C for 24 hours. The dried solid was milled, sieved, and stored in sample bottles for analyses.

A total of 24 experiments were conducted using four temperatures (200, 230, 255 and 270°C) and six B/W ratios (0.1, 0.18, 0.25, 0.43, 0.67 and 1.0.). A holding time of 20 minutes at peak temperature was used to minimize further mass loss [28]. Each experiment was conducted in triplicate.

### Analyses

The mass yield of hydrochar (MY [%]), the energy densification ratio (EDR [–]) and the energy yield (EY [%]) were calculated using Eqs (5) to (7):

$$MY=\frac{m_{hc}}{m_{b}}\times 100$$ (5)

$$EDR=\frac{HHV_{hc}}{HHV_{b}}$$ (6)

EY=MY×EDF (7)

where *m*_hc_ (kg) is the dry mass of the hydrochar, and *HHV*_hc_ (MJ/kg) and *HHV*_b_ (MJ/kg) are the higher heating values of the hydrochar and raw DM. The calorific values were determined using an OSK 200 bomb calorimeter (Ogawa Sampling, Saitama, Japan) by combusting at least 0.5 g of each sample in the calorimeter [29].

The ash content of each sample was determined using an electric muffle furnace (FUL220FA, ADVANTEC, Japan) by incinerating 1 g of oven-dried sample at 600°C for 3 hours. The volatile matter (VM) was determined using ASTM standard procedure E872 by heating the samples at 950°C for 7 minutes in an electric furnace. Then, the fixed carbon (FC [%] = 100 − VM [%] − ash [%]) and the fuel ratio (FR [–] = FC/VM) were calculated.

The carbon, hydrogen and nitrogen contents of the samples were determined using a CE-440 elemental analyser (Exeter Analytical, North Chelmsford, MA, USA). The oxygen content was calculated by difference (O [%] = 100 − C [%] − H [%] − N [%] − ash [%]). The percentage mass loss in carbon (*m*_c,loss_ [%]) and oxygen content (*m*_o,loss_ [%]) were calculated using Eq (8) by considering the absolute initial and final masses of the raw sample (*m*_*i*,b_[kg]) and the produced hydrochars (*m*_*i*,hc_ [kg]).


$$m_{i,loss}=\frac{m_{i,b}-m_{i,hc}}{m_{i,b}}\times 100\;(i=carbon\;or\;oxygen)$$ (8)

The combustion experiment was carried out using a thermal gravimetric analyser (TGA/DSC 1, Star System, Mettler Toledo, USA) under an air atmosphere. About 20 mg of sample was placed in an Al_2_O_3_ crucible and heated from 32 to 900°C with an air flow rate of 100 mL/min at a heating rate of 10°C/min. For each sample, the TGA experiment was repeated at least twice for accuracy [30]. The thermogravimetric (TG) and differential thermogravimetric (DTG) data were used to determine the following combustion parameters: ignition temperature (*T*_*i*_ [°C]), burnout temperature (*T*_*b*_ [°C]), burn out time (*B*_*t*_ [min]), residual mass (*R*_*m*_ [%]), maximum mass loss rate (*DTG*_*m*_ [%/min]) and its corresponding temperature (*T*_*m*_ [°C]). *T*_*i*_ indicates the temperature at which the fuel starts to burn while *T*_*b*_ denotes the temperature for complete combustion of the fuel; both were determined by the TG-DTG tangent method [30, 31]. *T*_*m*_ is the temperature at the maximum mass loss rate (DTG) or the peak temperature. The comprehensive combustion index (*CCI* [min^−2^°C^−3^]) and the combustion stability index (*CSI* [min^−1^°C^−2^]) were calculated using Eqs (9) and (10), respectively [30, 32, 33].


$$CCI=\frac{{DTG}_{m}\times {DTG}_{av}}{T_{i}^{2}\times T_{b}}$$ (9)

$$CSI=8.5875\times 10^{7}\times \frac{{DTG}_{m}}{T_{i}\times T_{m}}$$ (10)

## Results and discussion

### Classification of the HTC process by the degree of saturation

**Fig 2:**
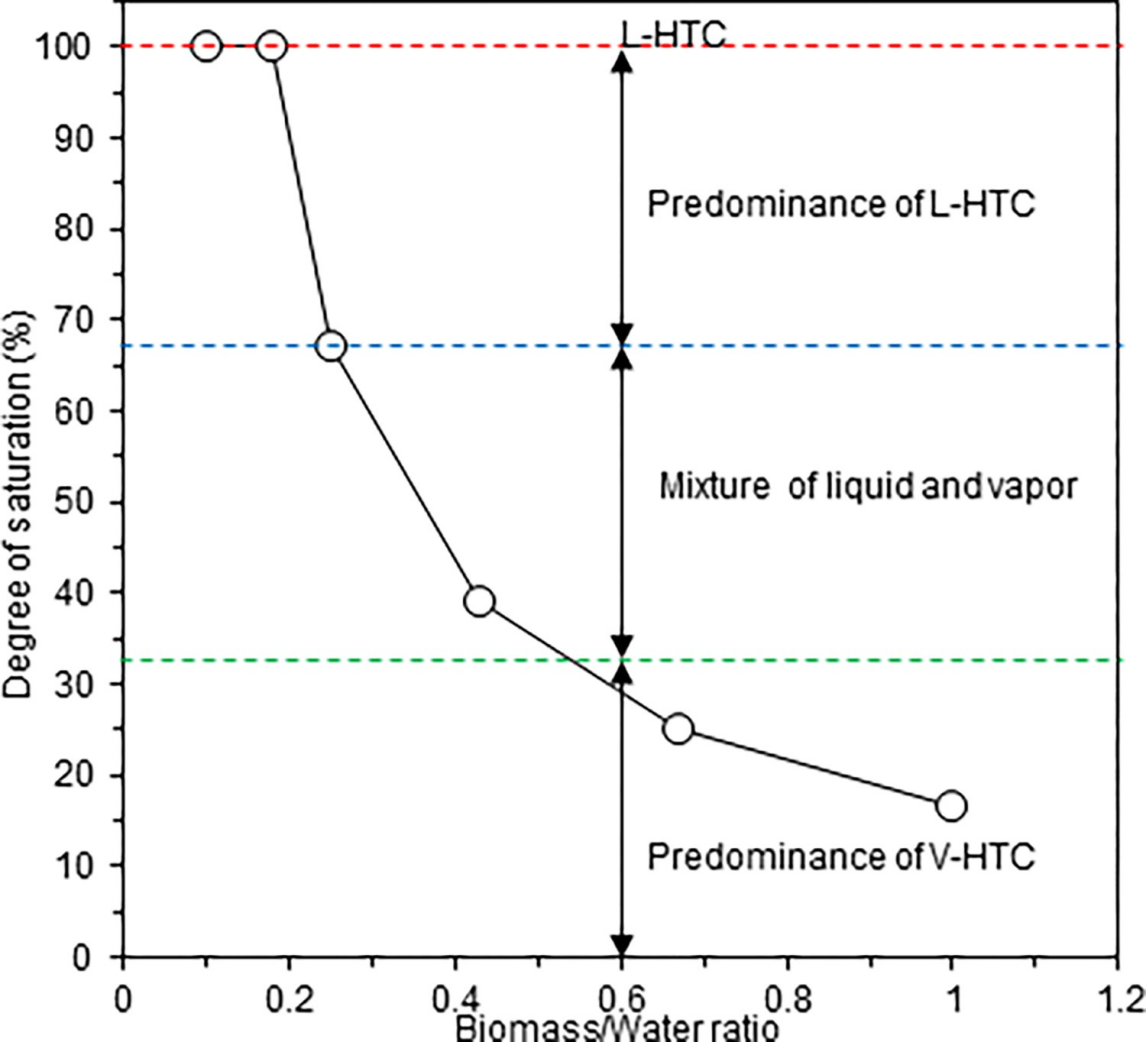
Classification of the HTC process based on the B/W ratio.

Fig 2 shows the degree of saturation (*S*_*r*_) based on the B/W ratio at initial conditions. According to the definition described in the Methods section, the HTC processes at each B/W ratio were largely complete for L-HTC at B/W ratios of 0.1 and 0.18, for L-HTC at a B/W ratio of 0.25, for a mixture of vapour and liquid phase at a B/W ratio of 0.43, and for V-HTC at B/W ratios of 0.67 and 1.0. At B/W ratios of 0.1 and 0.18 the raw feedstock was pasty, but little leaching of gravitational water from the prepared feedstock was observed. When the B/W ratio of the feedstock was further increased, the liquid-like properties of the feedstock changed to a plastic-like state at a B/W ratio of 0.43 and then to a semi-solid state at B/W ratios of 0.67 and 1.0. The determination of the degree of saturation and visual observation during feedstock preparation suggest an increase in the free air space where water vapour could easily penetrate the feedstock at higher B/W ratios.

### Mass yield

**Fig 3:**
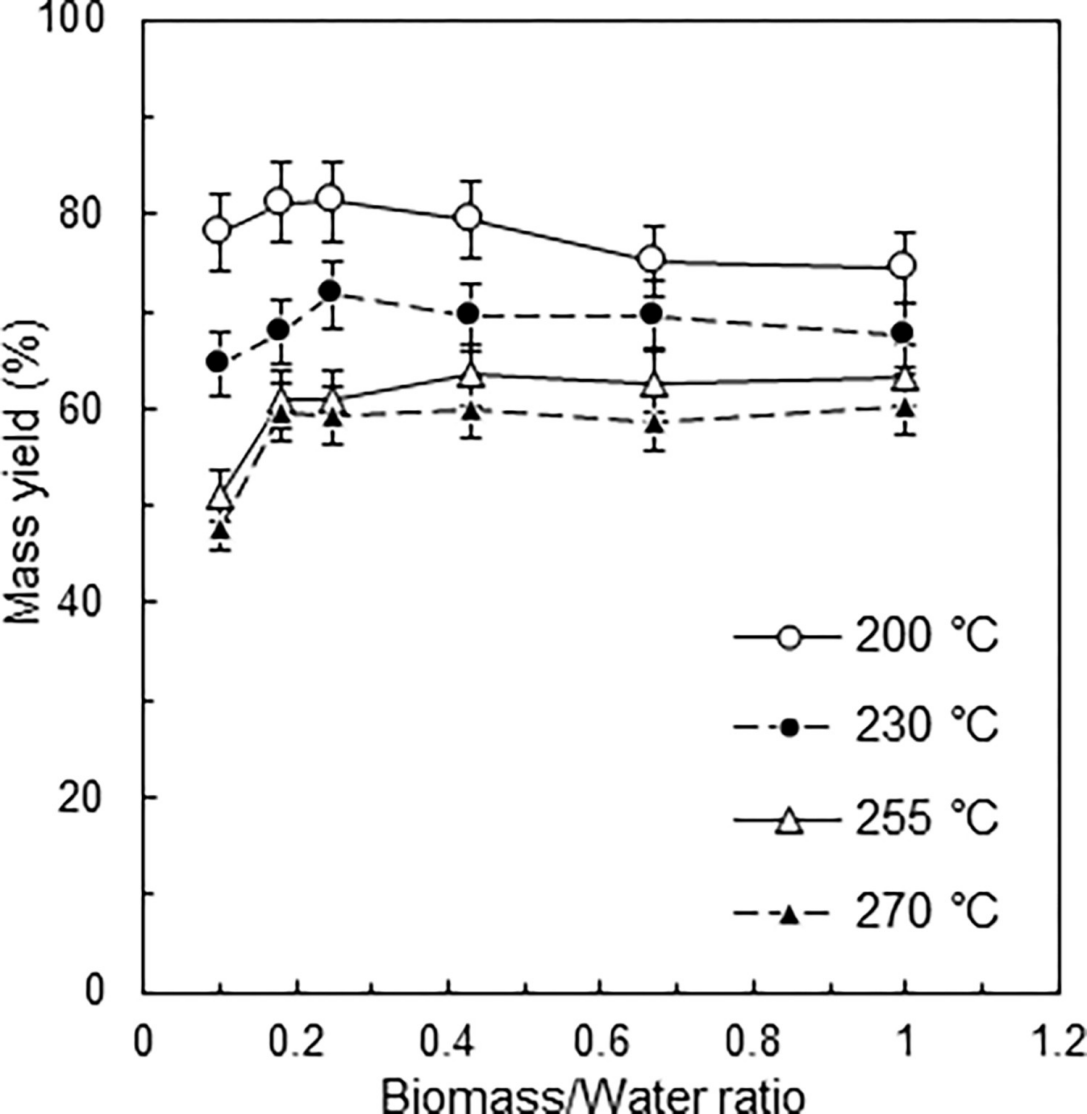
Mass yields at different B/W ratios and process temperatures.

Fig 3 shows the mass yield of hydrochar prepared at different temperatures and B/W ratios. The mass yields decreased with increasing process temperature. The mass yields showed only small fluctuations, regardless of the B/W ratio. The mass yield at the lower production temperatures (200 and 230°C) was the maximum at a B/W ratio of 0.25. At higher production temperatures (255 and 270°C), the mass yield increased at a B/W ratio of 0.18 and then became stable. These observed differences can be attributed to differences in reaction severity, since the mass yield in a thermochemical process indicates reaction severity. Consequently, higher temperature promotes biomass decomposition, accompanied by the elimination of carboxyl, carbonyl and hydroxyl groups, producing CO_2_, CO and H_2_O [34, 35]. At 255 and 270°C, the mass yield above a B/W ratio of 0.18 was higher than that at a B/W ratio of 0.1, due to either (i) the reaction severity decreasing above a B/W ratio of 0.18, resulting in higher mass yield, or (ii) the mass yield increasing due to physical deposition or chemical bonding of degraded substances on the hydrochar surface, although the reaction severity remains essentially stable above a B/W ratio of 0.18. Explanation (i) is based on liquid being superior to vapour for decomposing biomass, while explanation (ii) assumes there is little difference between each medium for decomposition, but degraded substances dissolved in the liquid phase are more likely to contact the hydrochar surface due to the higher B/W ratio. Previous studies suggest that the polymerization of dissolved substances in the liquid phase and subsequent precipitation of insoluble solid substances on the hydrochar are more prevalent at higher B/W ratios, thereby increasing mass yield [16, 19, 23]. Furthermore, proximate and ultimate analyses revealed that DM is exposed to a more severe reaction at higher B/W ratios (discussed below in Table 1). Given these considerations, the authors conclude that explanation (ii) is more likely.

**Table 1:** Proximate and ultimate analyses, and EDR of the hydrochars.

Sample name		Proximate analysis (wt.%)			Ultimate analysis (wt.%)				FR (-)	EDR (-)
Temp. (°C)	B/W ratio	VM	*FC	Ash	C	H	N	O*		
Raw	-	70.3±6.5	15.7±1.3	13.9±0.7	42.6±1.2	5.4±0.1	1.9±0.0	36.2±1.4	0.223	-
200	0.1	63.3±4.3	21.6±1.8	15.1±0,9	47.9±2.1	5.2±0.1	2.2±0.0	29.6±2.4	0.341	1.13
	0.18	62.6±7.9	21.3±2.0	16.1±0.5	47.8±1.5	5.2±0.0	2.1±0.0	28.8±1.1	0.340	1.14
	0.25	62.1±8.5	20.8±2.2	17.2±0.8	47.8±3.1	5.2±0.0	2.1±0.0	27.7±1.6	0.335	1.16
	0.43	61.6±6.9	24.2±1.1	14.3±0.3	50.7±2.2	5.3±0.2	2.1±0.0	27.7±1.8	0.393	1.23
	0.67	58.7±9.0	24.8±1.5	16.6±0.6	48.7±1.9	5.2±0.1	2.1±0.0	27.5±1.9	0.422	1.26
	1.0	58.9±5.8	25.2±0.9	15.9±1.4	53.8±1.8	5.2±0.0	2.4±0.0	22.7±0.9	0.428	1.31
230	0.1	63.4±8.1	21.5±1.3	15.1±0.9	50.2±2.5	5.2±0.2	1.9±0.0	27.6±1.1	0.339	1.18
	0.18	59.6±6.2	24.5±1.6	15.8±0.8	50.5±1.8	5.2±0.5	2.1±0.0	26.4±2.9	0.411	1.19
	0.25	60.2±5.3	22.9±0.8	16.9±1.1	49.5±1.2	5.3±0.3	2.4±0.1	25.9±1.2	0.380	1.19
	0.43	55.2±6.2	23.6±0.9	21.1±1.3	53.8±2.1	5.1±0.3	2.4±0.0	17.6±1.5	0.428	1.27
	0.67	54.8±4.5	24.9±1.9	20.2±0.6	53.2±1.9	5.1±0.3	2.4±0.0	19.2±1.7	0.454	1.30
	1.0	54.4±5.1	28.3±3.0	17.4±0.3	53.8±3.1	5.2±0.3	2.4±0.2	22.7±0.8	0.520	1.37
255	0.1	50.8±3.8	26.1±1.5	23.1±0.1	55.4±1.1	5.1±0.1	2.4±0.1	14.1±3.0	0.514	1.27
	0.18	51.9±3.9	26.0±3.0	22.0±1.0	54.1±1.2	5.1±0.2	2.5±0.0	16.3±1.8	0.501	1.30
	0.25	51.1±5.9	29.5±1.0	19.4±0.7	55.7±2.1	5.2±0.1	2.6±0.0	17.1±1.4	0.577	1.31
	0.43	49.1±3.8	28.5±2.0	22.4±1.3	52.8±1.9	5.0±0.0	2.8±0.1	17.0±1.1	0.580	1.30
	0.67	47.4±3.1	28.4±0.7	24.3±2.0	56.7±1.7	4.9±0.3	2.6±0.0	11.6±2.3	0.599	1.38
	1.0	49.5±7.1	31.8±1.3	18.7±0.4	58.5±2.3	5.1±0.2	2.7±0,0	15.1±1.0	0.642	1.43
270	0.1	49.7±6.2	29.6±3.1	20.7±0.9	53.9±2.8	4.9±0.3	2.6±0.1	17.9±0.6	0.596	1.28
	0.18	48.1±4.3	30.0±1.0	21.9±0.5	53.8±1.9	4.9±0.5	2.7±0.0	16.6±0.5	0.624	1.33
	0.25	50.6±4.7	28.9±4.0	20.4±0.6	55.1±2.5	5.1±0.4	2.7±0.2	16.7±0.4	0.571	1.34
	0.43	45.2±3.2	30.9±1.1	23.9±2.1	53.9±1.5	4.8±0.5	2.9±0.0	14.4±0.7	0.684	1.31
	0.67	44.6±5.0	33.9±1.6	21.5±1.8	59.6±2.0	4.9±0.2	2.8±0.0	11.2±0.3	0.760	1.46
	1.0	42.6±3.0	37.5±2.1	19.9±0.9	59.1±3.2	5.0±0.3	2.8±0.0	13.3±0.1	0.880	1.46

*Calculated by difference

### Energy analyses

**Fig 4:**
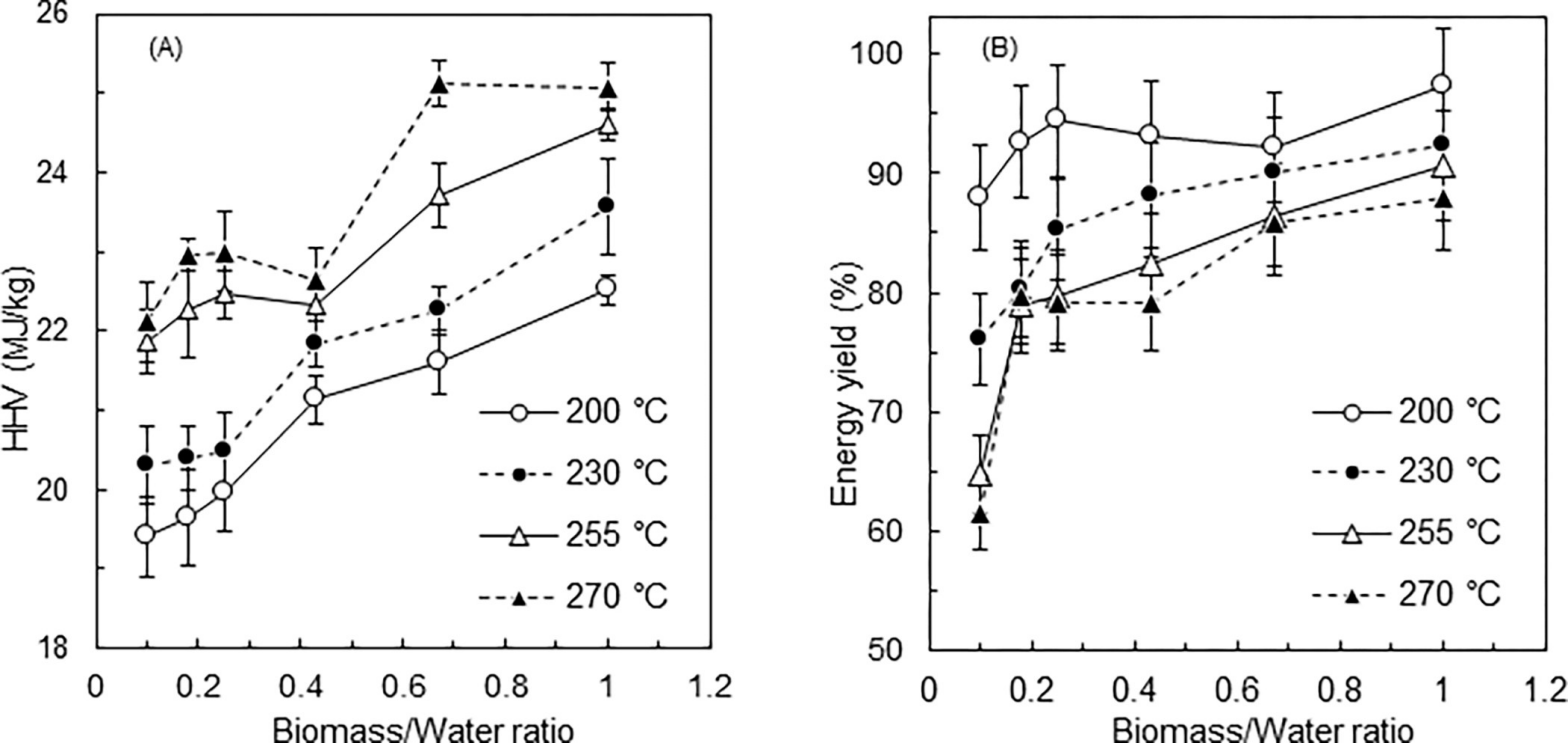
(A) Measured HHV and (B) EY at different B/W ratios and process temperatures.

The HHV of hydrochar increased from 17.2 to 25.1 MJ/kg as the B/W ratio and process temperature increased (Fig 4A). Higher temperatures increase hydrolysis, decarboxylation, dehydration, aromatization and re-condensation reactions, increasing the HHV of hydrochars [8, 11, 24]. The HHV showed upward trends with increasing B/W ratio, consistent with previous studies [5, 14, 23]. Given that deposition or bonding of degraded substances on the hydrochar surface increases at higher B/W ratios, as described in the previous section, these substances likely contribute to increasing the HHV of hydrochars. Indeed, Volpe and Fiori [23] found that a higher B/W ratio in the HTC of olive waste promotes the formation of secondary char, which has a relatively high carbon content, on the produced hydrochar, although the study used lower B/W ratios of 0.08 to 0.25 compared to the present study. Kambo et al. [18] reported that the recirculation of HTC process water, rich in organic acids (acetic, formic, levulinic and glycolic acid) due to biomass degradation, improves the HHV of hydrochar. Therefore, the increased severity of the reaction may be another reason for the increase in HHV, since the acid concentration is likely higher and the acidity higher in V-HTC, where the liquid content is lower.

The EDR was calculated to evaluate the degree of energy densification due to the process. As expected, the EDR trend was similar to that of the HHVs, with a maximum EDR of 1.46 observed at 270°C at a B/W ratio of 0.67 and at 270°C and a B/W ratio of 1.0 (Table 1). The use of V-HTC with increasing B/W ratio may achieve more effective energy densification compared to L-HTC. Previous studies conducting the HTC of DM at 240°C at a B/W ratio of 0.05 for 4 hours observed EDRs of 1.27 and 1.37, respectively [9, 11]. In contrast, the present study achieved an EDR of 1.37 at a lower temperature of 230°C and a shorter holding time of 20 minutes using a higher B/W ratio of 1.0.

The EY increased as the B/W ratio was increased across all the process temperatures tested but decreased with increasing process temperature (Fig 4B). In general, there is a negative correlation between the mass yield and the EDR: as the reaction severity increases, the mass yield decreases and the calorific value increases. The present study found that the EY increased with an increase in the B/W ratio, due to the mass yield not changing significantly, while the HHV increased with increasing B/W ratio (Figs 3 and 4A). Accordingly, the energy analysis indicated that V-HTC with a high B/W ratio has both a higher hydrochar production capacity (kg m^−3^ s^−1^) than L-HTC and can produce higher quality solid biofuel, a great advantage of V-HTC. The drop in the observed EY at 200°C at a B/W ratio of 0.67 is attributed to the drop in mass yield.

### Proximate and ultimate analyses of the hydrochars

The proximate and ultimate properties of the raw samples and produced hydrochars are presented in Table 1. Overall, the VM decreased and the FC and ash content increased with increasing temperature. For example, the VM decreased from 70.3±6.5% to 42.6±3.0% while the FC increased from 15.7±1.3% to 37.5±2.1% when the DM was hydrothermally treated at 270°C at a B/W ratio of 1.0. The decreased VM may have been converted to other substances during the process, such as liquid or gaseous products [27, 36], increasing FC. FR, which is the ratio of FC to VM, is used to rank fuel quality [37] and is thus increased by this process. The influence of the B/W ratio on the FR was more pronounced at higher B/W ratios across the process temperatures tested, indicating that higher quality hydrochars would be produced under V-HTC conditions. Indeed, the FR of the hydrochar at the predominantly V-HTC conditions of 270°C and a B/W ratio of 0.67 to 1.0 was higher than 0.6 (reported for lignite) and close to 1.2 (reported for subbituminous coal) [38, 39]. The ash content increased from 13.9% to 23.9% upon increasing the process temperature to 270°C. Wu et al. [9] also observed an increased ash content of DM, from 24.2% to 40.4%, by increasing the process temperature to 280°C, perhaps because ash in biomass increases upon further reduction in the VM at higher temperature [15]. In contrast, the effect of the B/W ratio on ash content showed an unexpected trend. In general, the ash content of hydrochar can decrease in some cases because the ash may leach into the liquid phase during HTC [28]. Therefore, it was expected that the highest ash content would be observed at a B/W ratio of 1.0, since the higher the B/W ratio, the less likely such ash leaching would occur. However, the results showed an increasing trend in ash content up to B/W ratios of 0.43–0.67 and then a decrease in ash content at a B/W ratio of 1.0, where the liquid phase was the smallest. Perhaps vapour is more effective at leaching ash than bulk water, although we cannot currently offer a clear explanation.

**Fig 5:**
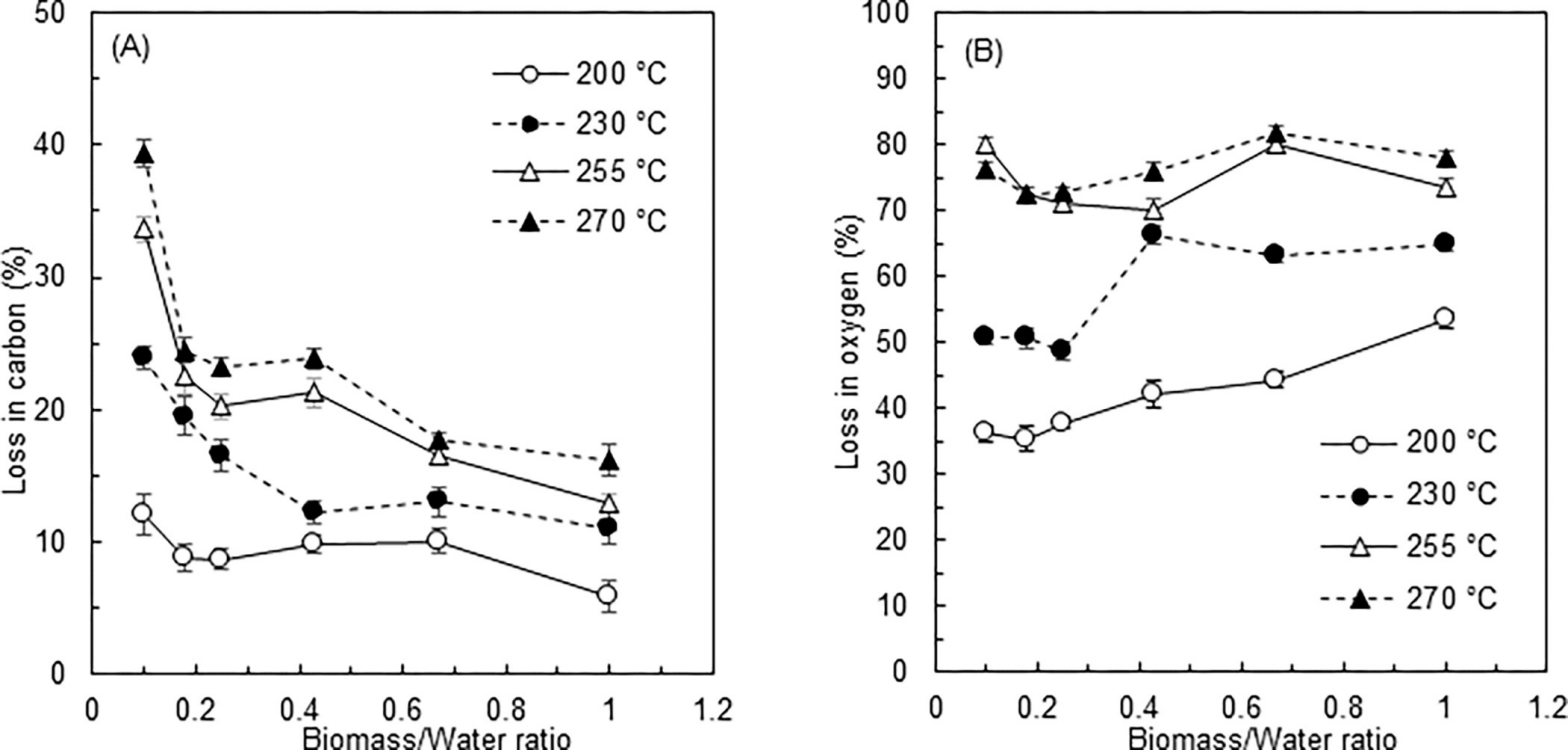
Losses in (A) carbon and (B) oxygen.

The ultimate analyses confirmed that the carbon content increased from 42.6±1.2% to 59.6±2.0% and the oxygen content decreased from 36.2±1.4 to 11.2±0.3% with increasing severity of the processing conditions. The change in hydrogen and nitrogen content was small: hydrogen decreased from 5.4% to 4.9% while nitrogen increased from 1.9% to 2.9%. Generally, oxygen in biomass is removed due to decarbonylation, decarboxylation and dehydration during the HTC process, and the degree of oxygen removal is pronounced at higher temperatures, increasing the relative carbon content of hydrochar [11, 40, 41]. This typical trend with increasing process temperature was observed in the present study. Notably, the increase in carbon and the decrease in oxygen appeared to be promoted at higher B/W ratios. To understand the increase and decrease of carbon and oxygen in more detail, their percentage losses are shown in Fig 5. The use of a higher B/W ratio, which corresponds to the transition from L-HTC to V-HTC, tended to decrease carbon loss, showing that V-HTC is superior for retaining carbon in the hydrochar compared to L-HTC. This suggests that degraded substances deposited or chemically bonded to the hydrochar surface contain more carbon: i.e., more secondary char is formed in a V-HTC environment. The oxygen content of degraded substances is generally relatively high but oxygen is removed through dehydration or carboxylation before interaction with hydrochar, resulting in secondary char formation on hydrochar. More oxygen was removed with increasing B/W ratio at process temperatures of 200°C and 230°C but was little affected by the B/W ratio at higher temperatures of 255°C and 270°C. Thus, the impact of B/W ratio on deoxygenation is higher in mild HTC processes but the process temperature increases oxygen removal in severe HTC processes.

**Fig 6:**
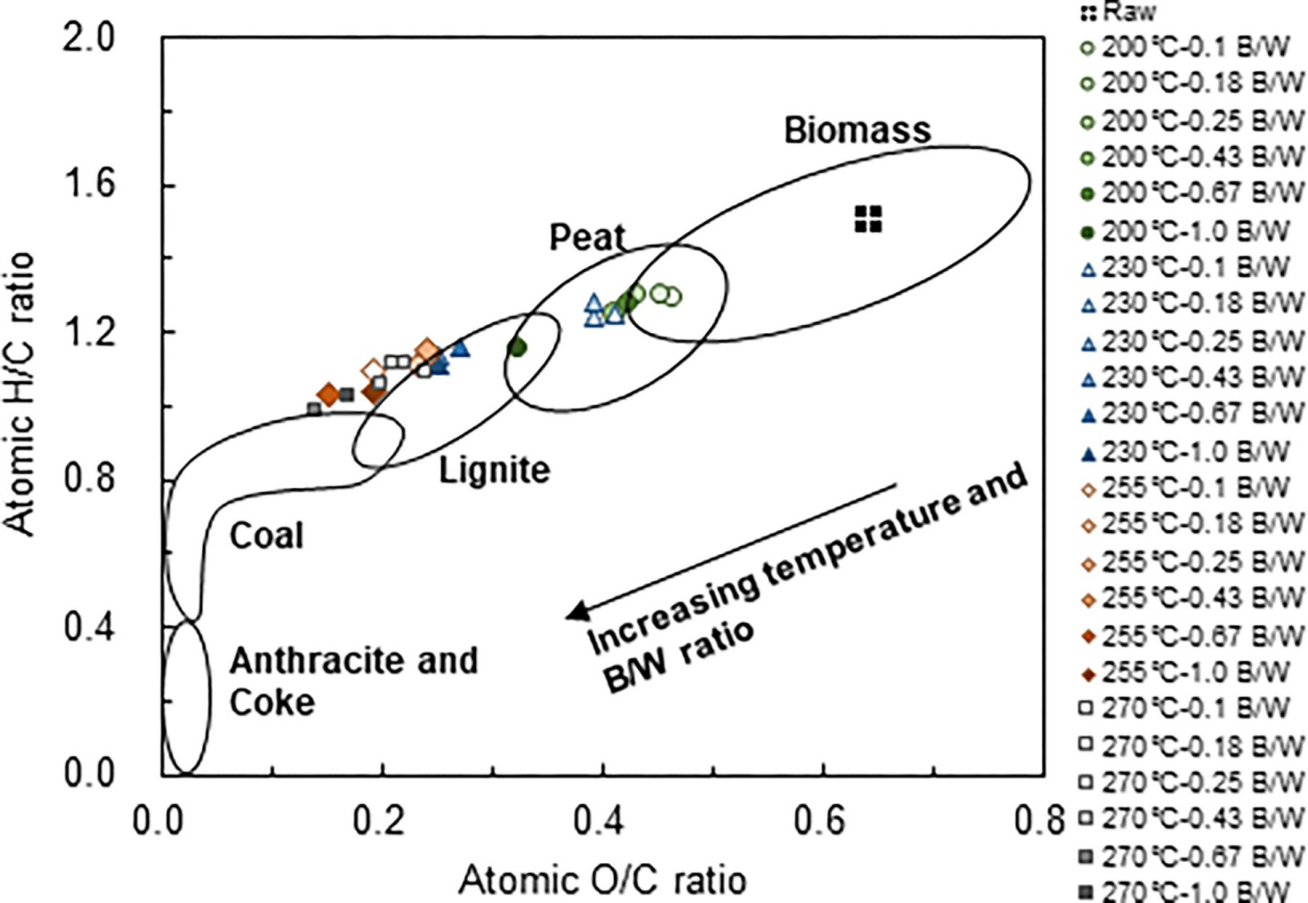
van Krevelen diagram for the hydrochars.

Data from the ultimate analyses were used to plot the van Krevelen diagram (Fig 6), which provides general information about the quality and type of fuel and alterations in biomass composition. A fuel with low atomic ratios of O/C and H/C is highly preferred because it produces less smoke, water vapour, and energy loss during combustion [17, 35, 42]. The diagram showed that the degree of coalification of the hydrochars increased as the temperature and B/W ratio increased. Thus, increasing the B/W ratio from 0.1 to 1.0 at each temperature is effective for decreasing the O/C and H/C ratios of the raw feedstock. At a given temperature, the use of a higher B/W ratio gave lower atomic ratios of O/C and H/C, showing that a higher quality solid biofuel can be produced through predominantly V-HTC conditions rather than L-HTC. For example, hydrochars produced at between 200°C at a B/W ratio of 0.1 up to 230°C at a B/W ratio of 0.43 were peat-like, hydrochars produced at 230°C at a B/W ratio of 0.67 up to 270°C at a B/W ratio of 0.43 were lignite-like, and hydrochars produced at 255°C at a B/W ratio of 0.43 and at 270°C at B/W ratios of 0.67 and 1.0 were coal-like.

### Thermal behaviour and combustion parameters

**Fig 7:**
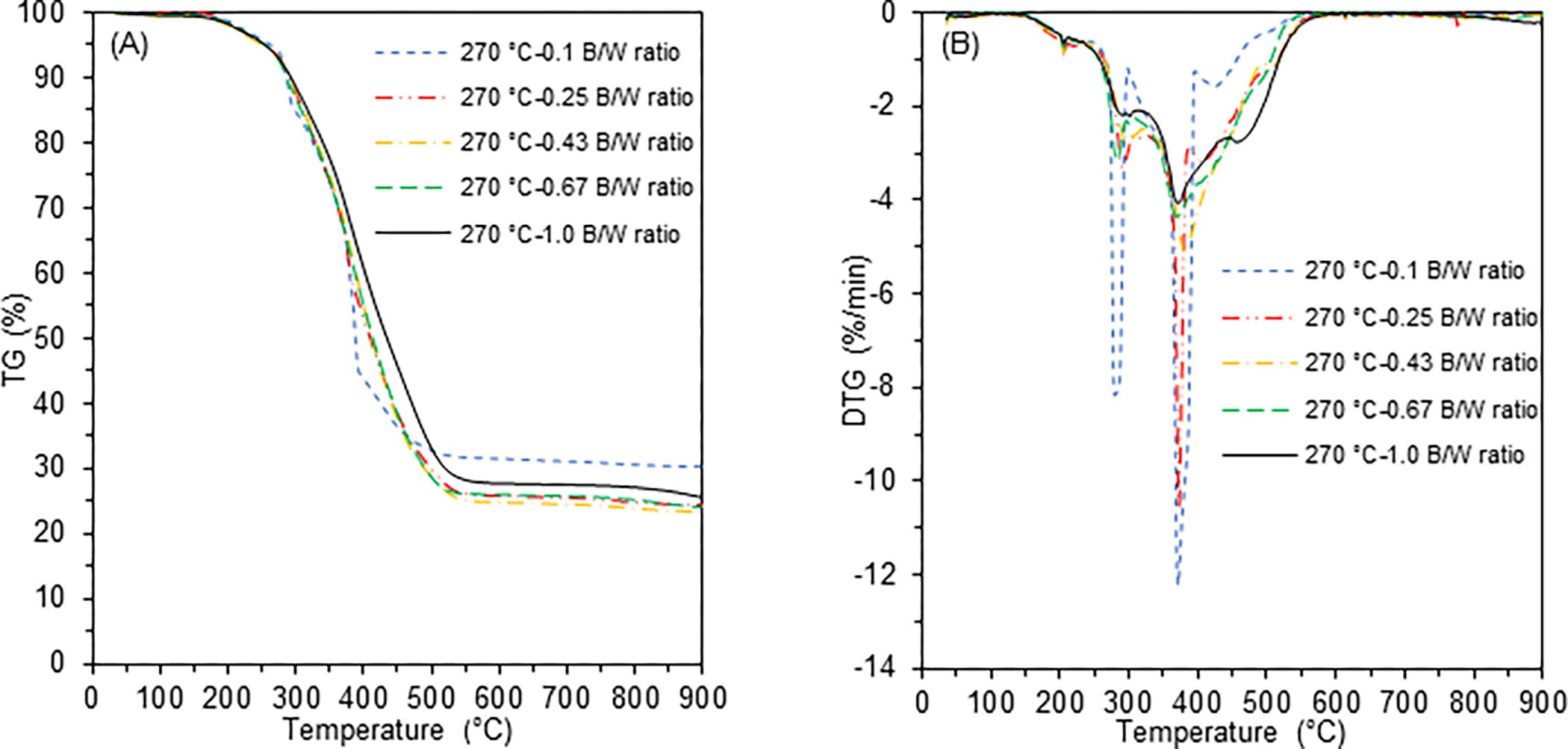
(A) TG for hydrochars at 270°C (B) DTG for hydrochars at 270°C.

Fig 7 shows the TG and the DTG curves for the hydrochars produced at 270°C using a heating rate of 10°C/min. As shown in Fig 7B, regardless of the B/W ratio, four distinctive peaks were observed corresponding to the loss of moisture (50 to 200°C), low molecular weight volatiles (200 to 300°C), lignin decomposition (300 to 400°C), and char combustion (450 to 600°C) [30, 38]. The decomposition peaks tended to decrease with increasing B/W ratio and the smallest peak was observed at 270°C and a B/W ratio of 1.0, probably due to this hydrochar having the lowest VM and highest FC (Table 1). Progressive reduction in the decomposition peaks at higher B/W ratios indicates enhanced thermal stability of the hydrochar [43].

The ignition temperature (*T*_*i*_) is an important combustion parameter to determine the probability of fire or explosion when using a hydrochar as a solid biofuel [43, 44]. The hydrochar produced at 270°C and a B/W ratio of 0.1 has the lowest *T*_*i*_ while the hydrochar produced at 270°C and a B/W ratio of 1.0 has the highest *T*_*i*_, as shown in Table 2. The increase in *T*_*i*_ is due to reduction of the VM content in the hydrochar during the process [43]. Thus, the safety of hydrochars during handling, storage and transportation as solid biofuels increases as the B/W ratio is increased [38, 45]. On the other hand, the CCI and CSI decreased with increasing B/W ratio, suggesting that the combustibility of hydrochars produced under V-HTC is lower than those produced under L-HTC. However, excessive VM increases the CCI and CSI, and therefore may cause an unstable flame and combustion, leading to high heat loss [37]. Thus, combustion performance of the prepared hydrochars should be explored in more detail in future studies.

**Table 2:** Combustion parameters determined from the TGA curve at 10°C/min (270°C).

Sample	*T*_*i*_	*T*_*m*_	*DTG*_*m*_	*DTG*_*av*_	*T*_*b*_	*B*_*t*_	*R*_*m*_	*CCI*	*CSI*
	(°C)	(°C)	(%/min)	(%/min)	(°C)	(min)	(%)	(min^−2^ × °C^−3^)	(min^−1^ × °C^−2^)
0.1 B/W	218.8	354.7	-12.1	-0.8	511.7	50	29.6	3.9 × 10^−7^	1.3 × 10^4^
0.25 B/W	223.5	371.8	-10.6	-0.9	555.5	51	24.4	3.4 × 10^−7^	1.1 × 10^4^
0.43 B/W	223.4	380.7	-5.1	-0.9	563.9	52	23.3	1.6 × 10^−7^	5.2 × 10^3^
0.67 B/W	223.5	372.5	-4.3	-0.9	564.2	52	24.0	1.4 × 10^−7^	4.4 × 10^3^
1.0 B/W	223.8	374.7	-3.9	-0.9	573.3	54	25.3	1.2 × 10^−7^	3.9 × 10^3^

*T*_*i*_: ignition temperature; *T*_*m*_: peak temperature; *DTG*_*m*_: the maximum mass loss rate; *DTG*_*av*_: average mass loss rate; *T*_*b*_: burn out temperature; *B*_*t*_: burn out time; *R*_*m*_: residual mass; *CCI*: comprehensive combustion index; *CSI*: combustion stability index

## Conclusion

This study investigated the hypothesis that hydrochar with improved fuel properties would be produced from low moisture content biomass without the need to add water. To verify the hypothesis, DM at high and low moisture conditions was hydrothermally treated using L-HTC and V-HTC conditions. The results showed that the V-HTC process is superior to the L-HTC process in improving the fuel properties of DM. The V-HTC process is conducted at a higher B/W ratio, where the lower liquid content may facilitate the formation of secondary char on the hydrochar surface and increase the severity of the reaction due to the higher acid content, resulting in higher energy densification and mass yield. As a result, the V-HTC process is expected to have a higher hydrochar production capacity and require less water compared to the L-HTC process. Proximate analysis revealed that the ash content was at maximum at B/W ratios of 0.43 and 0.67 rather than 1.0, and thus further studies are required to understand this behaviour. The obtained results nonetheless support the hypothesis of the study.

## Supporting information

S1 Table Average mass yield, HHV, energy yield and energy densification ratio. Average values used to plot the graphs for the mass yield, HHV and EDR at increasing B/W ratio.
(DOCX)
