# Supplementary material for: Improvement of the fuel properties of dairy manure by increasing the biomass-to-water ratio in hydrothermal carbonization
Source: PLoS One. 2022 Jul 18;17(7):e0269935. doi: 10.1371/journal.pone.0269935 (PMC9292100; doi:10.1371/journal.pone.0269935)
Supplement: S1 Table — Average values used to plot the graphs for the mass yield, HHV and EDR at increasing B/W ratio. (DOCX) [file pone.0269935.s001.docx]

**Improvement of the fuel properties of dairy manure by increasing the biomass-to-water ratio in hydrothermal carbonization**

Mohammed Aliyu^1,2^, Kazunori Iwabuchi^3,*^, Takanori Itoh^4^

^1^ Graduate School of Agriculture, Hokkaido University, Kita 9, Nishi 9, Kita-ku, Sapporo, Hokkaido 060-8589, Japan

^2^ Agricultural and Bioresources Engineering, Federal University of Technology, P. M. B. 65, Minna, Niger State, Nigeria

^3^ Research Faculty of Agriculture, Hokkaido University, Kita 9, Nishi 9, Kita-ku, Sapporo, Hokkaido 060-8589, Japan

^4^ Tanigurogumi Corporation, Shiobara 1100, Nasushiobara, Tochigi 329-2921, Japan

^*^Corresponding email: [iwabuchi@bpe.agr.hokudai.ac.jp](mailto:iwabuchi@bpe.agr.hokudai.ac.jp)

| **Temperature (°C)** | **Biomass/Water ratios** | **Mass Yield (%)** | **HHV (MJ/Kg)** | | **Energy yield (%)** | **ED ratio** |
| --- | --- | --- | --- | --- | --- | --- |
| 200 | 0.1 | 78.01 | 19.41 |  | 87.98 | 1.127833 |
|  | 0.18 | 81.18 | 19.64 |  | 92.64 | 1.141197 |
|  | 0.25 | 81.32 | 19.98 |  | 94.41 | 1.160953 |
|  | 0.43 | 75.77 | 21.14 |  | 93.07 | 1.228356 |
|  | 0.67 | 73.37 | 21.61 |  | 92.13 | 1.255665 |
|  | 1.0 | 74.40 | 22.52 |  | 97.36 | 1.308542 |
| 230 | 0.1 | 64.53 | 20.31 |  | 76.15 | 1.180128 |
|  | 0.18 | 67.85 | 20.39 |  | 80.39 | 1.184776 |
|  | 0.25 | 71.70 | 20.48 |  | 85.32 | 1.190006 |
|  | 0.43 | 69.39 | 21.84 |  | 88.06 | 1.26903 |
|  | 0.67 | 69.63 | 22.27 |  | 90.10 | 1.294015 |
|  | 1.0 | 67.50 | 23.57 |  | 92.44 | 1.369553 |
| 255 | 0.1 | 50.99 | 21.87 |  | 64.80 | 1.270773 |
|  | 0.18 | 60.93 | 22.28 |  | 78.88 | 1.294596 |
|  | 0.25 | 60.99 | 22.47 |  | 79.63 | 1.305636 |
|  | 0.43 | 63.50 | 22.34 |  | 82.43 | 1.298083 |
|  | 0.67 | 62.74 | 23.72 |  | 86.47 | 1.378268 |
|  | 1.0 | 63.40 | 24.60 |  | 90.62 | 1.429402 |
| 270 | 0.1 | 47.85 | 22.12 |  | 61.50 | 1.285299 |
|  | 0.18 | 59.71 | 22.97 |  | 79.69 | 1.334689 |
|  | 0.25 | 59.25 | 23.00 |  | 79.18 | 1.336432 |
|  | 0.43 | 60.11 | 22.64 |  | 79.08 | 1.315514 |
|  | 0.67 | 58.73 | 25.13 |  | 85.76 | 1.460198 |
|  | 1.0 | 60.40 | 25.08 |  | 88.02 | 1.457292 |

**S1 Table.** **Average mass yield, HHV, energy yield and energy densification ratio**
